# A Multi-Method Approach to a Comprehensive Examination of the Psychiatric and Neurological Consequences of Intimate Partner Violence in Women: A Methodology Protocol

**DOI:** 10.3389/fpsyt.2021.569335

**Published:** 2021-02-19

**Authors:** Tara E. Galovski, Kimberly B. Werner, Katherine M. Iverson, Stephanie Kaplan, Catherine B. Fortier, Jennifer R. Fonda, Alyssa Currao, David Salat, Regina E. McGlinchey

**Affiliations:** ^1^Women's Health Sciences Division, National Center for Posttraumatic Stress Disorder, Veterans Affairs Boston Healthcare System, Boston, MA, United States; ^2^Department of Psychiatry, Boston University School of Medicine, Boston, MA, United States; ^3^Women's Health Sciences Division, National Center for PTSD, VA Boston Healthcare System, Boston, MA, United States; ^4^The Translational Research Center for Traumatic Brain Injury and Stress Disorders and the Geriatric Research, Education and Clinical Center, Veterans Affairs Boston Healthcare System, Boston, MA, United States; ^5^Department of Psychiatry, Harvard Medical School, Cambridge, MA, United States; ^6^Martinos Center for Biomedical Imaging, Massachusetts General Hospital, Charlestown, MA, United States

**Keywords:** intimate partner violence, traumatic brain injury, women, post-traumatic stress, concussion

## Abstract

The number of women in the United States that experience blows to the head during assaults by intimate partners is substantial. The number of head blows that result in a traumatic brain injury (TBI) is virtually unknown, but estimates far exceed numbers of TBI in parallel populations (e.g., blast exposure, accidents, sports) combined. Research on the impact of TBI on post-traumatic stress disorder (PTSD) in survivors of intimate partner violence (IPV) is sparse. This methodology paper describes the comprehensive, multi-method approach used by a multi-disciplinary team of investigators from several different fields of expertise to assess the interaction of psychiatric, cognitive, psychological, and physical conditions that result from IPV. Using state-of-the-art instruments, a comprehensive assessment of lifetime trauma exposure, lifetime history of TBI, psychiatric history, and a full assessment of current cognitive, neuropsychological and biomedical function was conducted with 51 female survivors of IPV who screened positive for PTSD. This multi-method assessment included clinician-administered diagnostic interviews modified to specifically assess the sequelae of IPV, standardized self-report surveys, neuropsychological tests, structural, diffusion, and functional neuroimaging and blood-based biomarkers. The specific details and full report of the results of the full study are beyond the scope of this methodology paper. Descriptive characteristics of the complex clinical presentation observed in this unique sample are described. The sample reported high rates of trauma exposure across the lifespan and 80% met full criteria for current PTSD. Women also reported high rates of lifetime subconcussive head injury (88.2%) and TBI (52.9%) from various etiologies (35.3% secondary to IPV). Descriptive findings from the methodological protocol described here have begun to reveal information that will advance our understanding of the impact of subconcussive head injury and TBI on recovery from mental injury among IPV survivors.

## Introduction

Intimate partner violence (IPV), defined as physical violence, sexual violence, stalking or psychological aggression by a current or former intimate partner, is a public health crisis in the United States (1, 2). Approximately 1 in 3 adult women (34%) experience physical IPV during their lifetime, with 1 in 4 women (23%) experiencing severe physical IPV such as being hit with a fist or hard object, strangled, beaten, or assaulted with a weapon (3). Although women and men both experience IPV, women are more likely than men to experience severe physical IPV and, subsequently, incur more injuries (3, 4). IPV-related injuries are often repeated over time (5, 6). Injuries to the head, neck, and face are most frequently described and, indeed, are reported by 35–94% of IPV survivors (7). Approximately 50% of IPV survivors report attempted strangulation at the hands of a partner (8), adding to the potential for damage to the brain secondary to anoxia.

While physical injury secondary to IPV is common, psychiatric consequences are substantial as well. Post-traumatic stress disorder (PTSD) is one of the most common psychiatric diagnoses secondary to exposure to the trauma of IPV (9). Women who experience physical IPV are 2.3 times more likely to have PTSD compared to those who do not experience physical violence from a partner (10). PTSD is characterized by re-experiencing a traumatic event followed by persistent avoidance of trauma-related stimuli, negative cognitions and mood, and persistent symptoms of increased arousal (11). Estimates of PTSD following IPV range from 31 to 63.8% depending on the sample composition and assessment methodology (9, 12).

The complexity of the relationship between head injury and PTSD secondary to IPV is apparent in a growing literature (13–16). In order to develop interventions to successfully promote recovery, it is imperative to understand the potential synergistic interaction of the full spectrum of relevant neurobiological, psychiatric, psychological, social, and environmental risk factors that contribute to functional, physical and mental health outcomes. The universe of moderating variables that can contribute to the observed complex clinical presentations in the IPV population is immense. Understanding how negative physical, functional, and mental health outcomes evolve over time requires a comprehensive assessment of both the physical and psychological impacts of IPV and consideration of the larger context of prior injuries and the cumulative effect of lifetime exposures to traumatic events. This methodology paper describes the comprehensive, multi-method approach used by a multi-disciplinary team of investigators from several different fields of expertise to assess the interaction of psychiatric, cognitive, psychological, and physical conditions that result from IPV.

### The Complexity of Assessment of Head Injuries and PTSD in IPV

The preponderance of studies in the IPV literature to date have focused primarily on one or two physical and/or mental conditions that result from IPV or have controlled for these conditions, but have not considered them more comprehensively as primary outcomes. For example, as pointed out by Valera et al. (17), studies focusing on PTSD among female IPV survivors typically exclude or do not consider head injuries in their design. This is problematic because symptomatology that has historically been linked to post-traumatic stress secondary to IPV may be better understood by considering the interaction of head injury, unique physiological disruptions (e.g., anoxia), environmental factors, and the chronic stress characteristics of IPV (18). Contrarily, executive functioning deficits that are more characteristic of head injuries such as mild traumatic brain injury (mTBI) [e.g., (19)], including cognitive, behavioral, and emotional difficulties, may be better attributable to PTSD or other comorbid psychiatric conditions (e.g., disruptions in sleep, irritability or aggression, disinhibition, anxiety, depression) (11). Disentangling this complex clinical presentation and arriving at differential diagnoses requires a comprehensive assessment critical in informing treatment. This methodology paper describes the most comprehensive biopsychosocial assessment of the effects of IPV assaults to date.

**Figure 1:**
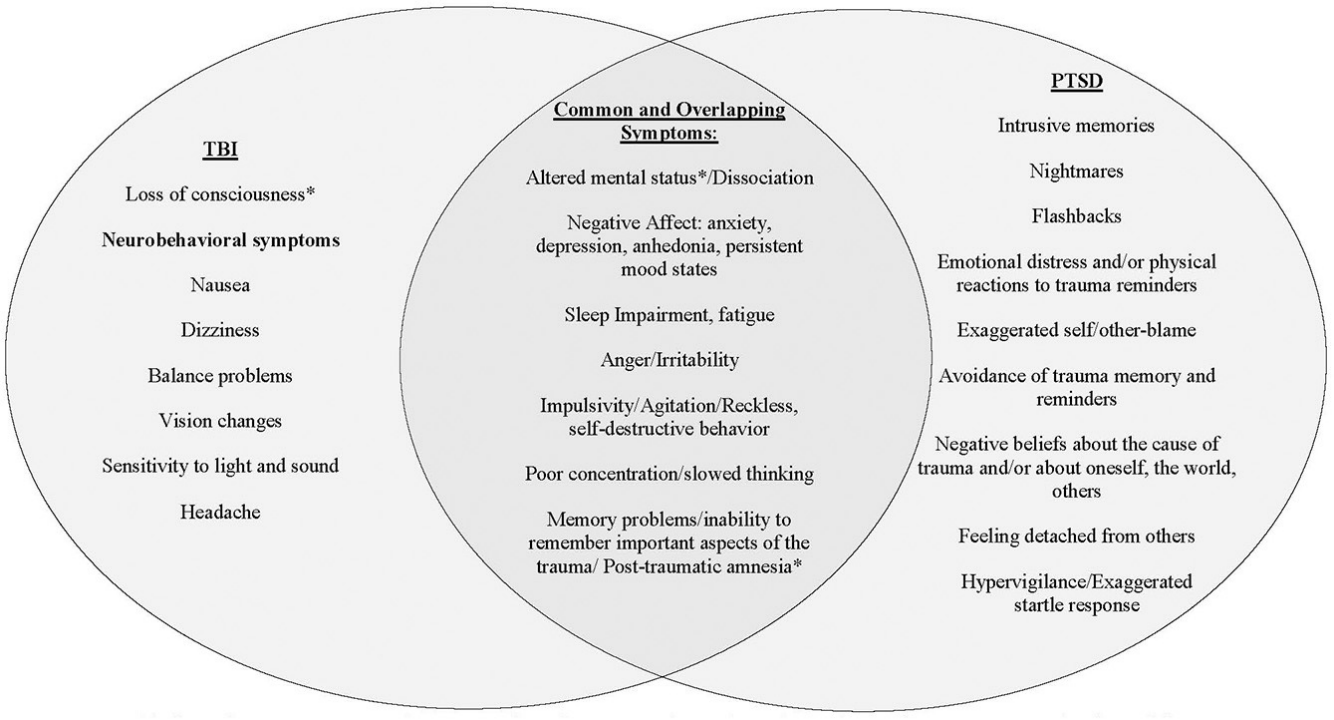
Discrimination of symptoms associated with TBI from those associated with PTSD. This figure is meant as a general representation of constructs for each syndrome, but each symptom can arise from different underlying mechanisms whether neurologic or psychiatric. The asterisk (^*^) indicates diagnostic symptoms of acute TBI. Common symptoms of TBI and PTSD can be almost identical and therefore very difficult to distinguish the etiology. These symptoms require a thorough clinical evaluation to establish a temporal relationship between traumatic event, physical injury, symptom onset, and course.

As noted, injuries to the head, neck and face are the most common type of injury reported during IPV assaults (7) and some percentage of those injuries will result in a traumatic brain injury (TBI). TBI is defined as a physiological disruption in brain function resulting from a blow to the head (20, 21). The three primary acute symptoms of TBI are alteration in mental status (AMS), post-traumatic amnesia (PTA), and loss of consciousness (LOC). The severity of TBI is further specified as mild, moderate or severe and determined by the duration of the acute symptoms. Mild TBI (mTBI) accounts for nearly 80% of all TBIs (22). Accurate diagnosis of TBI requires careful assessment of each of these three acute symptoms with particular care to differentiate physiological and psychological responses (i.e., acute stress symptoms, dissociation, disorientation and/or confusion from the experience of a frightening or life-threatening event) that can occur during and after the injury (23). See Figure 1 for an overview of the significant overlap between symptoms of TBI and PTSD that contributes to the diagnostic complexity of disentangling symptoms consistent with physical injury from those better attributable to mental injury.

The preponderance of research examining subconcussive head injury and TBI has focused primarily on injury secondary to falls, motor vehicle accidents, combat-related concussive blasts, and sports-related injuries (24, 25). However, estimates suggest that ~23 million women in the US have experienced a TBI from IPV (26), numbers far exceeding estimates of TBI in military and athlete cohorts combined (17, 27). Findings from these parallel, head-injured populations are difficult to generalize to injuries sustained during IPV assaults because of the nature of the blow to the head, neck, and face and the context in which the injury occurs (16, 17). For example, the majority of head blows during IPV assaults are quite variable in nature. Blows can be either focal or diffuse, can include primary rotational blows (e.g., punches to the face and head) and/or secondary blunt force blows (e.g., being thrown into a wall or down stairs or being hit by a hard object such as a bat). Violence typically increases in frequency and severity over time (28). This escalating pattern of assault, often with little time for healing between injuries, can lead to both substantial and obvious injury as well as subconcussive head injury that can go undetected, unreported, undiagnosed, and untreated among IPV survivors (8, 29–31), [e.g., (32)]. In addition to blows to the head, strangulation is particularly unique to IPV and increases the possibility of brain injury due to anoxia. Anoxic brain injury (ABI) occurs when the brain is deprived of oxygen. This deprivation affects medial temporal and subcortical regions of the brain [e.g., basal ganglia, hippocampus, limbic structures, (33)], and, if prolonged, damage can become more diffuse. Experiencing both blows to the head and strangulation during violent intimate partner relationships is not uncommon and results in compounded risk for brain injury. In a study examining injuries among women seeking help following IPV, ~75% of participants reported having been strangled and nearly 50% reported repeated blows to the head (34).

The assessment of head injury and diagnosis of TBI (including anoxic brain injury) in the IPV population is clearly complicated. This complexity contributes to the lack of consensus in reported prevalence of TBI, with rates ranging from 28 to 100% (6, 14, 26, 35). The considerable variability across studies is due, in part, to inconsistencies in definitions and terminology used to describe the same condition [e.g., mTBI, concussion, and head injury, (36)] and further complicated by the wide range of instruments used to assess TBI including screening items, self-report questionnaires, and clinician-administered diagnostic interviews (37). These methodological limitations in the larger TBI literature may be particularly apparent in studies with IPV samples which tend to rely on general screening items or scales to estimate TBI vs. validated screening and clinician-administered diagnostic instruments (26, 27).

### Domains of Assessment and Differential Diagnoses

#### Diagnosing TBI

Arriving at an accurate diagnosis of TBI is an essential first step in understanding its impact on PTSD. The Boston Assessment of Traumatic Brain Injury, Lifetime (BAT-L) (23) is a validated, reliable, and comprehensive semi-structured clinical interview to characterize and diagnose mild TBI across the lifespan. The BAT-L was selected for the current study due to its strong psychometric qualities, its ability to sensitively distinguish between subconcussive head injury and TBI, and to differentiate alterations in acute symptoms of TBI (AMS, PTA, LOC) from other common physiological and psychological reactions to injury and trauma. For the purposes of this study, the BAT-L was expanded to specifically query head blows and injuries across participants' IPV relationship(s) (38).

#### Lifetime and Current Trauma History

The context in which injuries associated with IPV assaults occurs also warrants specific attention in a comprehensive assessment. Assaults by intimate partners happen, by definition, in a chronically traumatizing and invalidating environment. This controlling environment is characterized by emotional abuse and betrayal, coercion, isolation, and lack of independence or ability to access resources (39, 40). The psychiatric and psychological effects of the traumatic assaults and abuse interact with the physical and neurological effects of the head injury and amplify the overall clinical presentation of IPV survivors (16). Finally, an assessment of the psychological sequelae of IPV must also consider the effects of previous trauma exposures. Prior history of abuse in both childhood and adulthood (in addition to IPV) are prevalent among women who experience IPV (41–43) and contribute to the cumulative trauma burden and current post-traumatic stress. A thorough assessment of the full history of violence within intimate partner relationships and non-IPV trauma across the lifespan is necessary to inform diagnosis and to specify treatment decisions.

#### Psychiatric Comorbidity

The extent to which the diffuse symptoms associated with comorbid psychiatric conditions such as PTSD, depression, anxiety, and substance use disorders; psychological difficulties such as sleep disturbances, impulsivity, aggression or irritability, dissociation; and behavioral changes such as social isolation and withdrawal overlap can contribute to the difficulties in arriving at differential diagnoses (25, 44, 45). Within the IPV literature, the range of comorbid psychiatric conditions and TBI has been documented in several studies, revealing strong associations between IPV-related TBI and PTSD, depression, anxiety, insomnia, and poorer overall perceptions of mental health (13–15, 46). A full psychiatric and psychological assessment is critical in evaluating the effects of IPV on mental health.

#### Neuropsychological Functioning

Further complicating assessment and treatment in this population is some evidence of possible neuropsychological impairments among women who experience IPV, but it remains unclear to what extent TBI and PTSD may contribute to or account for these problems. For example, cognitive deficits, including poorer working memory, visuoconstruction, and executive function have been observed among women with IPV, including among those with and without PTSD (47). Yet in a sample of women with PTSD secondary to IPV, Twamley et al. (48) observed that higher PTSD severity was associated with slower processing speed, and higher dissociation symptoms were associated with poorer reasoning performance. It is notable that these studies did not attend to TBI history, which is important for future research as there is some evidence that TBIs in the context of IPV are associated with impairment in neuropsychological functioning including memory deficits, difficulty in learning, and poor cognitive flexibility (6, 49). Based on research to date, it remains unclear what role PTSD and TBI (and other psychiatric/psychological factors) play in these associations. Given that the cognitive domains implicated in TBI overlap with cognitive deficits secondary to PTSD, interpretation of the results of a full neuropsychological assessment is critical in disentangling the physical and psychological contributions to the full clinical presentation (50).

#### Neurological Signature of TBI

Neuroimaging of markers of processes hypothesized to be implicated in impairment associated with PTSD and TBI provides additional clues about the neuropsychological and neurobiological consequences of brain injury. Alterations in neural connectivity and/or the detection of structural abnormalities can then be mapped onto performance on neuropsychological tests. In the larger TBI literature, there is evidence to suggest that the role of the TBI in poor psychiatric and psychosocial outcomes is substantial; however, most studies to date fail to determine the independent contribution of TBI(s) in the onset and propagation of these problems (51). As described, several studies suggest that psychiatric distress (PTSD and depression) and not injury–related characteristics, is uniquely associated with reported neurobehavioral symptoms following head injury [e.g., (50, 52, 53)]. On the other hand, as discussed, TBIs in the context of IPV are associated with impairment in neuropsychological functioning including memory deficits, difficulty in learning, and poor cognitive flexibility (6, 49). These cognitive impairments purportedly arise from diffuse axonal injuries in brain networks that are important for attention, memory, and executive function (54–56), suggesting that observed impairment is independently associated with the brain injury. These distinctions are important in informing interventions.

To date, only two published studies have examined the neurological signature of TBI specifically in an IPV population. Utilizing resting state functional magnetic resonance imaging and neuropsychological measures, Valera and Kucyi (56) examined IPV-related head injuries (including TBI, anoxic brain injuries, and subconcussive blows), neural connectivity, and cognitive functioning in 20 women IPV survivors. Seventy-five percent of participants had suffered IPV-related, repetitive head injuries. Severity of brain injury was associated with reduced connectivity within the salience network, the neural network positively associated with memory and learning (16, 17). This effect remained even after controlling for psychiatric distress (e.g., PTSD and depression symptoms). In a follow-up study, researchers examined micro-structural neurologic changes and cognition in the same sample with IPV-related mTBI. Overall brain injury severity scores were negatively associated with fractional anisotrophy in the posterior and superior corona radiata. However, no association was found between the cognitive measures of learning, memory, and cognitive flexibility and fractional anisotrophy in these regions (16, 17). Although Valera and colleagues statistically controlled for PTSD symptoms, further research is needed to assess the independent and interactive effects of the brain injury and PTSD on mental and physical health outcomes and related functional impairments.

#### Blood-Based Biomarkers

Finally, a major gap in the literature is the consideration of biomedical data in understanding the effects of IPV. Understanding the physical health history and assessing basic health indicators including blood pressure, height, weight, body mass index, and pulse can identify risk factors as well as negative health outcomes associated with IPV. In particular, Valera et al. (17) argue that blood-based biomarkers are a critical area for IPV research. For example, the authors note that tau and amyloid beta are candidate biomarkers for this population because they have been implicated in the development of TBI-related cognitive and neurodegenerative disorders (17). There is also evidence of elevated concentrations of tau in service members and Veterans with concurrent mild TBI and PTSD (57). Furthermore, a recent review indicated that there are significant alterations in neurotransmitter, peptide, and steroid hormone levels in PTSD (58). To our knowledge, no studies have specifically examined these or other blood-based biomarkers among women IPV survivors with PTSD nor whether there are additive effects of TBI with concurrent PTSD among this population.

### Current Study

The current study sought to describe the methodology used in the most comprehensive, multi-method assessment of the long-term effects IPV to date. The methodology outlined in this paper positions this unique, multi-disciplinary investigative team to accomplish the overarching goal of understanding the impact of head injuries and TBI on PTSD in an all-female sample of IPV survivors. Specifically, in future publications, we seek to characterize the relative contributions of TBI and/or subconcussive head injury to psychiatric, psychological, and functional outcomes in this population with the ultimate goal of identifying novel targets for intervention and enhancing the overall effectiveness of current, single modal treatment strategies. The results of those study aims are too broad to present in this methodology paper. Here, we describe the methodology used in this comprehensive assessment and present the clinical descriptives of the study sample, but we will not include findings from the neuroimaging, blood-based biomarkers, full range of psychiatric comorbidities, or neuropsychological assessment as those methodological details and analyses are beyond the scope of this paper.

## Methods

### Participants

**Figure 2:**
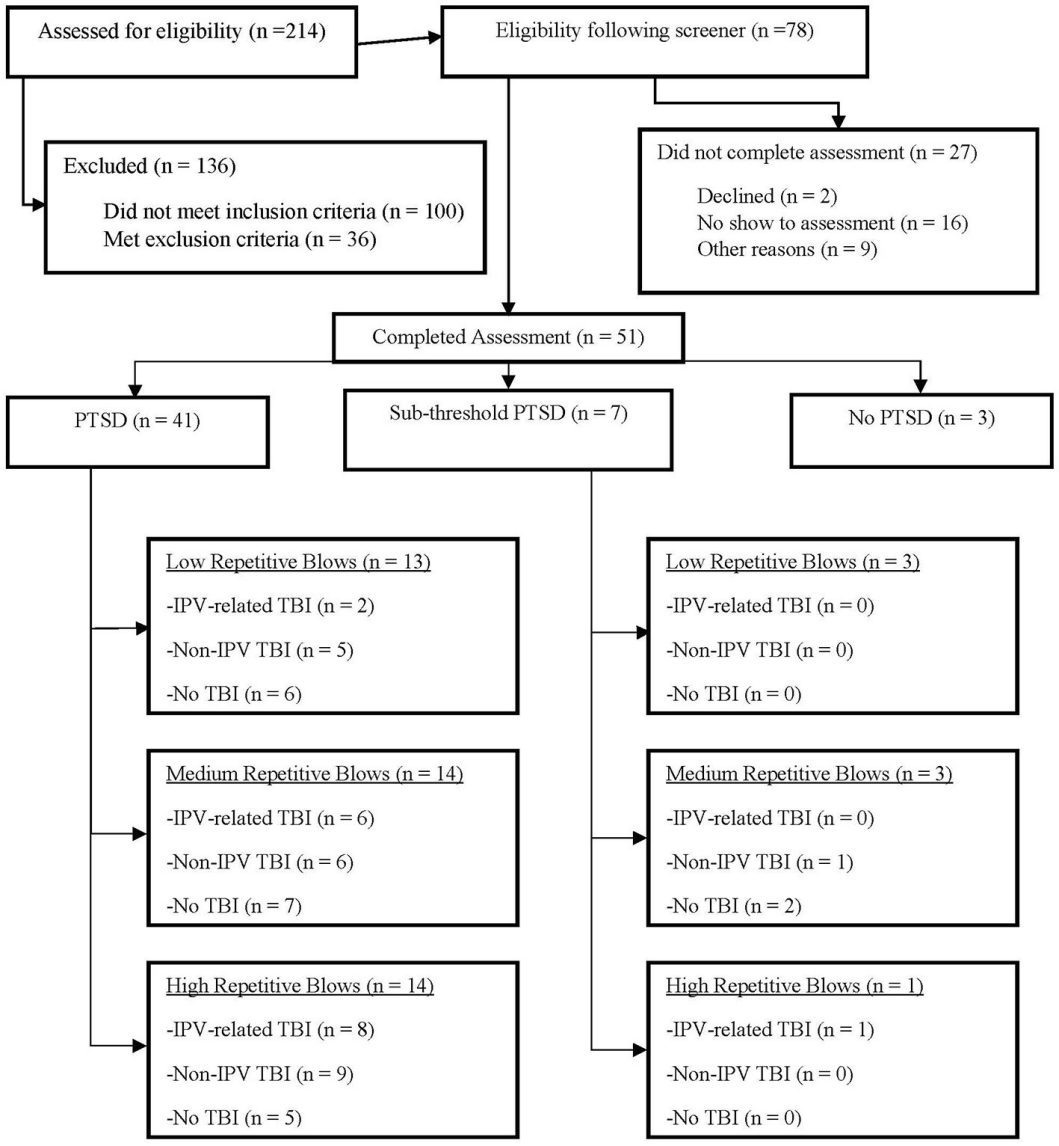
CONSORT diagram of study participants. The CONSORT flow diagram of the participants through the phases of assessment, further differentiated by PTSD status. The diagram represents low, medium, and high head injury rates for those participants who both meet for a full PTSD diagnosis, as well as those with subthreshold PTSD. PTSD, Post-Traumatic Stress Disorder; TBI, Traumatic Brain Injury. Other reasons for not completing the assessment after being found eligible include changes to the participants condition prior to or on the day of consent: bipolar disorder, active psychosis, chemotherapy, peritraumatic experience, pregnancy, moved out of state, multiple sclerosis.

Women were recruited from a mid-size, midwestern metropolitan area via flyers sent to agencies that serve survivors of IPV and through advertisement on social media. Women between the ages of 18–45 years old who reported a history of IPV and screened positive for probable PTSD on the PTSD Checklist for DSM-5 [PCL-5; (59)] screener during phone intake were invited to participate in the study, as these were the study's inclusion criteria. Exclusion criteria included history of neurological illness (e.g., Huntington's, Parkinson's, dementia, Multiple sclerosis), history of seizure disorders unrelated to head injury(ies), current diagnosis of Bipolar I disorder, schizophrenia or other psychotic disorders, and current active homicidal and/or suicidal ideation with intent requiring crisis intervention. Pregnancy and metal in the body were additional exclusion criteria, as the study design included MRI. Two hundred and fourteen women were screened by phone for the study. Of these, 136 were excluded from participation during the phone screen for the following reasons: 67 were over age 45, 24 did not meet screening cutoffs for probable PTSD, 5 denied a history of IPV, 4 lived out of state, 20 had Bipolar I Disorder, 3 reported a seizure disorder, 9 were pregnant, and 4 had metal in her body. Per these criteria, 78 women screened eligible. Twenty-seven of the 78 women did not complete the study for a variety of reasons including: 2 declined to participate after phone screen, 16 did not show up for scheduled assessments, 2 presented with symptoms consistent with exclusionary diagnoses on the day of assessment (Bipolar I and psychosis respectively), 2 were found to be pregnant on the day of the assessment, 1 moved out of state prior to assessment, 1 was in a developing traumatic situation and needed to decline participation, 1 was diagnosed with Bipolar Disorder by a physician prior to assessment, and two reported medical complications that prohibited participation prior to assessment (multiple sclerosis and beginning chemotherapy, respectively). The final sample included 51 participants (see Figure 2).

### Procedures

The study team consisted of a multidisciplinary partnership with expertise across relevant clinical and methodological domains and included investigators from the Women's Health Sciences Division of the National Center for PTSD, Translational Research Center for TBI and Stress Disorder (TRACTS), University of Missouri – St. Louis Missouri Institute of Mental Health (UMSL), and Washington University (WASHU) Center for Clinical Imaging Research. Once screened, participants signed informed consent. Clinical diagnostic interviews, neuropsychological assessment, and completion of psychological and psychosocial standardized instruments were conducted at UMSL. Biological data was collected, and imaging was conducted at WASHU. Assessments took ~12 h over the course of 2 days; participants were offered remuneration for their time ($150 for day 1 and $125 for day 2). The study was conducted under the oversight of Institutional Review Boards at UMSL, WASHU, and the VA Boston Healthcare System.

### Measures

#### Clinical Interviews

A master's level clinical assessor conducted the clinician-administered interviews to assess for trauma, PTSD, psychiatric comorbidity, subconcussive head injury, and TBI. Each case was reviewed by at least three doctoral-level psychologists (study investigators with relevant expertise in diagnosing TBI and psychiatric disorders) to achieve a consensus diagnosis. Interviews took ~4 h to conduct, though time was not limited and varied somewhat depending on number of traumas reported, psychiatric diagnoses, and head injuries.

##### Trauma Exposure

Lifetime exposure to traumatic events was assessed via a locally constructed clinician-administered interview (60). This interview captured participants' trauma history across the lifespan, history of intimate relationships with a specific focus on violence within those relationships, injuries sustained during assaults by intimate partners, and utilization of resources following IPV (e.g., health care, law enforcement). This information was supplemented with a battery of standardized self-report measures, a complete list of which can be found in Table 1.

**Table 1:** Self-report measures.

**Domain/Test**	**Abbreviation**	**Description**	**References**
**Traumatic Exposure and Trauma Symptoms**			
Women's Experience with Battering	WEB	Measures level of psychological vulnerability experienced in intimate relationships (i.e., perceptions of vulnerability to danger, loss of control to partner, disempowerment)	(61)
Composite Abuse Scale	CAS	Measures four dimensions of abuse: Severe Combined Abuse, Emotional Abuse, Physical Abuse, and Harassment	(62)
Conflict Tactics Scale	CTS-2	Assesses five ways in which conflict can be resolved in an intimate relationship, including: negotiation, physical aggression, physical assault, sexual coercion, and injury	(63)
PTSD Checklist DSM-5	PCL-5	Assesses DSM-5 symptoms of PTSD	(59)
Childhood Trauma Questionnaire	CTQ	Retrospective assessment of childhood abuse and neglect. Five clinical scales include: physical, sexual, emotional abuse, and physical and emotional neglect.	(64)
**Psychological Distress Psychosocial Impairment**			
Post-Traumatic Cognitions Inventory	PTCI	Measure of trauma-related thoughts and beliefs. Three subscales include: Negative Cognitions About the Self, Negative Cognitions About the World, and Self-Blame.	(65)
Neurobehavioral Symptoms Inventory	NSI	Measure of post-concussive symptom severity, includes three subscales: somatic/sensory, affective, cognitive	(66)
Pittsburgh Sleep Quality Index	PSQI	Measure of sleep quality that produces global score and seven subscale scores, including: sleep quality, sleep onset latency, sleep duration, sleep efficiency, sleep disturbances, use of sleeping medication, and daytime dysfunction	(67)
Depression and Anxiety Stress Scale	DASS-21	Produces three subscale scores to assess for severity of distress related to depression, anxiety, and stress	(68)
Brief Pain Inventory Short Form	BPI-SF	Assesses the severity of pain and the impact of pain on daily functions	(69)
State Trait Anger Expression Inventory	STAXI	Measure of the experience, expression, and control of anger. The measure consists of six primary subscales, including State Anger, Trait Anger, Anger-In, Anger-Out, Anger Control, and Anger Expression	(70)
Quality of Life Inventory	QOLI	Provides global measure of life satisfaction based on average of satisfaction ratings across a range of life functions	(71)
Satisfaction with Life Scale	SWLS	Global measure of life satisfaction using broad appraisal of life without differentiating between different domains	(72)
Fagerstrom Test for Nicotine Dependence	FTND	Measures personal history of current and chronic cigarette use and/or use of smokeless tobacco products	(73)
World Health Organization Disability Assessment Schedule 2	WHODAS-2	Measure of impairment due to health-related problems experienced in the past month. Assesses across six domains and includes general disability score.	(74)
**Sensory Functioning**			
Hearing Handicap Inventory for Adults	HHIA	Assess emotional, social/situational, and occupational reactions to hearing loss	(75)
Tinnitus Handicap Inventory	THI	Quantifies impact of tinnitus on daily living. 25 questions divided into 3 subgroups: functional, emotional and catastrophic.	(76)
Vertigo Symptom Scale Long Form	VSSL	Quantifies vertigo severity and somatic anxiety symptoms.	(77)
Dizziness Handicap Inventory	DHI	Measures the handicapping effects of vestibular dysfunction across three subscales: physical, emotional, and functional factors of dizziness-related handicap.	(78)

##### Assessment of PTSD

The Clinician Administered PTSD Scale for DSM-5 (CAPS-5) was used to assess for PTSD. The CAPS-5 is considered the gold-standard in PTSD assessment (79) for diagnosing current (past month) and lifetime PTSD. Weathers and colleagues (80) reported on the psychometric properties of the CAPS-5 in male combat veterans demonstrating strong interrater reliability (κ = 0.78–1.00, depending on the scoring rule) and test–retest reliability (κ = 0.83), as well as strong correspondence with a diagnosis based on the CAPS for DSM–IV (CAPS-IV; κ = 0.84 when optimally calibrated). CAPS-5 total severity score demonstrated high internal consistency (α = 0.88) and interrater reliability (ICC = 0.91), good test–retest reliability (ICC = 0.78), and good convergent validity with total severity score on the CAPS-IV (*r* =0.83). Inter-rater reliability via an external doctoral-level CAPS expert for CAPS scoring consensus was performed [Cohen's κ = 0.70 (current); 0.75 (lifetime)].

##### Assessment of Psychiatric Comorbidity

The Structured Clinical Interview for DSM-5 Disorders (SCID-5) (81) is a semi-structured interview used to diagnose lifetime and current (past-month) DSM-5 psychiatric disorders. Interrater reliability for SCID-5 diagnoses have been found to be good to excellent with kappa coefficients ranging from 0.59 to 1.00 (82). SCID-5 diagnoses have also displayed adequate internal consistency (Cohen's α = 0.78–0.97) (83). The following modules of the SCID were administered: Module A: mood disorders: bipolar I disorder, bipolar II disorder, major depressive disorder, persistent depressive disorder; Module B/C: psychotic screening; Module E: alcohol use disorder, substance use disorders; Module F: anxiety disorders: panic disorder, agoraphobia, social phobia, specific phobia, obsessive-compulsive disorder, generalized anxiety disorder (current only); Module I: feeding and eating disorders.

##### Assessment of Subconcussive Head Injury and TBI

The current study utilized an adapted form of the BAT-L, which has demonstrated validity and reliability for assessment of subconcussive head injury and TBI across the lifespan (23). This semi-structured interview is designed to obtain a detailed account of the respondent's lifetime history of head injuries. The semi-structured interview establishes a detailed timeline and gathers contextual information for events occurring before, during, and after the injury to estimate the duration of acute TBI symptoms (AMS, PTA, LOC) and determine TBI severity. The BAT-L was originally developed to assess blast exposure and TBI in military samples (23) and has demonstrated excellent psychometric properties, including correspondence with the Ohio State University TBI Identification Method (κ = 0.89; Kendall *b* = 0.95), and excellent interrater reliability (κ > 0.80) (23). A particular strength of the BAT-L is its use of a forensic approach to gather in-depth contextual information to differentiate change in mental status (AMS/PTA/LOC) vs. vision/hearing difficulty or psychological distress (including dissociation) associated with the IPV event in order to increase accuracy of TBI diagnosis.

For this study the BAT-L was adapted by the study team to include a comprehensive assessment of potential IPV-related head injuries (both subconcussive head injury and TBI) and strangulation, referred hereafter to the BAT-L/IPV. This investigation was particularly interested in the effects of head injuries on associations between data elements. For the purposes of the current study, and to ensure stringent operationalization of the impact of specific type of injury we are assessing, we use the term “head injury” to describe injuries with the potential to cause neurobiological damage. Specifically, these include injuries resulting from assault by an intimate partner and include blunt force to the head (e.g., hit/punched in the face with fist, weapons or objects), head injuries resulting from falls and shoves, striking head on walls and furniture, and strangulation. We are not including other common IPV-related injuries to the face and neck that would not cause neurobiological damage (e.g., those that result from cutting, knifing, or burning) in our definition of head injury.

The BAT-L/IPV determined: (1) estimated lifetime incidence of IPV-related subconcussive head injury; (2) whether such injuries met criteria for IPV-related TBI; (3) lifetime incidence of IPV-related subconcussive head injury; (4) whether such injuries met criteria for lifetime non-IPV TBI; (5) lifetime incidence of strangulation; and (6) whether such injuries resulted in LOC. The incidence and severity of TBIs is based on American Congress of Rehabilitation Medicine standards and Department of Defense (DoD) criteria (see Figure 1); mTBIs, or concussion, are further subdivided into grade I, II, or III injuries (20, 84, 85). Finally, the BATL/IPV allows investigators to assess and detect the effects of cumulative subconcussive head injury (that did not meet diagnostic criteria for TBI) on outcomes. In summary, this instrument is critical in accurately diagnosing TBI and categorizing more heterogenous subconcussive head injury. Following administration of the BAT-L/IPV diagnostician should have a comprehensive clinical picture of the timing, number, developmental period, and severity of TBI and head injury across the lifetime and be able to estimate the cumulative effects of subconcussive blow both lifetime and within and IPV relationship. Interrater reliability of the IPV BAT-L/IPV in this study is strong (κs = 0.89; 38).

#### Self-Report Measures

To capture the extent of the experience of trauma and the impact on psychological, psychosocial and physical health indicators, a number of valid and reliable self-report measures were included in the battery to assess anger, health status, experiences with trauma, psychopathology, quality of life, sleep, and disability. Participants completed each measure during her assessment session. The complete list of measures and their descriptions are provided in Table 1.

#### Neuropsychological Functioning Assessment

The neuropsychological assessment battery included a comprehensive approach to assess cognitive functioning targeted to potential impairment associated with PTSD and TBI. The battery included assessments of premorbid abilities, attention, executive functioning, working memory, verbal learning and memory, psychomotor function, and symptom validity using both standard clinical neuropsychological tests as well as more precise computerized cognitive neuroscience measures (see Table 2). In addition to measurement considerations, such as the validity and reliability of tests, practical consideration (i.e., time demand and availability of alternate forms) guided test selection. We also implemented measures from the NIH Toolbox Cognitive Battery to include common data element recommendations by the NIH.

**Table 2:** Neuropsychological function assessment.

**Measure**	**Domain/construct**	**References**
Wechsler Test of Adult Reading (WTAR)	Premorbid verbal IQ	(86)
Green Verbal Medical Symptom Validity Tests (V-MSVT)	Validity	(87)
Gradual Onset Continuous Performance Task (GradCPT)	Attention and executive function: inhibition	(88)
Delis Kaplan Executive Function Scale (DKEFS) Color-Word Interference Test	Executive function: inhibition	(89)
Delis Kaplan Executive Function Scale (D-KEFS) Trail Making Test 2,4, and 5	Attention, psychomotor speed, executive functioning: sequencing, set-shifting	(89)
Iowa Gambling Test (IGT)	Executive functioning: decision making	(90)
California Verbal Learning Test-II (CVLT-II)	Verbal learning and memory	(91)
NIH Toolbox Cognition Battery		(92)
Picture Vocabulary	Memory (episodic)	
Flanker Inhibitory Control and Attention	Attention and executive function: inhibition	
List Sorting Working Memory	Memory (working)	
Dimensional Change Card Sort	Verbal fluency	
Pattern Comparison Processing Speed	Attention and executive function: cognitive flexibility	
Picture Sequence Memory	Executive function: processing	

#### Neuroimaging

As a primary aim of the current study was to understand the effects of PTSD and head injury on the brain structure, function, and connectivity following IPV, neuroimaging methodologies including structural, diffusion, and resting state functional imaging were included in our assessment (see Table 3). Consistent with the TRACTS protocol (93), we used a 3T Siemens Prisma MRI for image acquisition. Two whole-brain high-resolution images were acquired for each individual. These images were averaged for each participant to create a single image with high contrast-to-noise. Total imaging time was ~90 min for each scan. The MRI sequences and data processing assess properties of cortical and subcortical gray matter, microstructural integrity of the cerebral white matter, resting state networks, and functional connectivity.

**Table 3:** Neuroimaging assessment.

**Domain/Test**	**Measure**	**Sequence**
Structural morphometry	2X 3D T1-weighted MPRAGE Run 1	Flip Angle 7 deg, TE 3.35 ms, TR 2,530 ms, Slice Thickness 1.0 mm, In-Plane resolution 1.0 x 1.0 mm, Acquisition Time (TA) 6:02
Structural connectivity	2D Diffusion AP and PA	60 directions, b value=700, Flip Angle 90 deg, TE 103 ms, TR 10,000 ms, Slice Thickness 2.0 mm, In-Plane resolution 2.0 x 2.0 mm, TA 12:12
Functional Neuroimaging	BOLD Resting State Run 1	Flip Angle 90 deg TE 30 ms TR 3,000 ms Slice Thickness 3.0 mm FOV 192 mm In-Plane Resolution: 3 x 3 mm, TA 6:06
	BOLD Resting State Run 2	Parameters copied from BOLD Resting State Run 1
	BOLD Resting State Field Map	Flip Angle 90 deg TE 30 ms TR 3,000 ms Slice Thickness 3.0 mm FOV 192 mm In-Plane Resolution 3 x 3 mm, TA 4.5s
	Spin Echo Field Maps AP and PA	Flip Angle 90 deg TE 58 ms TR 3,500 ms Slice Thickness 3.0 mm FOV 192 mm In-Plane Resolution 3 x 3 mm, TA 0:14
Additional Scans	T2-weighted 3D FLAIR	Flip Angle 120 deg, TE 388 ms, TR 6,000 ms, Slice Thickness 1.0 mm, In-Plane Resolution 0.49 x 0.49 mm, TA 7:02
	T2-weighted 3D SPACE	Flip Angle 120 deg, TE 284 ms, TR 3,200 ms, Slice Thickness 1.0 mm, In-Plane Resolution 1.0x 1.0 mm, TA 4:46
	3D Susceptibility-weighted imaging (SWI)	Flip Angle 15 deg, TE 20 ms, TR 27 ms, Slice Thickness 1.20 mm, In-Plane Resolution 1.2 x 1.2 mm, TA 4:24

#### Blood-Based Biomarkers

Due to a dearth of literature focused on blood-based biomarkers for PTSD and TBI in an IPV sample, participants were asked to fast for 12 h prior to their blood being drawn and a certified phlebotomist drew 80 milliliters of blood from each participant. All staff and personnel handling blood samples received proper safety training overseen by the UMSL Environmental Health and Safety Department. Though a complete list of all blood analyses exceeds the scope of this paper, it is important to note that assays included in the current study were consistent with overall indicators of heath, as well as neuroendocrine and immune markers previously found to be associated with PTSD and TBI in other populations (e.g., Cortisol, Brain-derived neurotrophic factor, Neuron-specific enolase, Interleukin −10 & −6, and Tau). After samples were collected, a portion were processed (centrifuged and aliquot) and transferred to Quest Diagnostics Inc. to generate data on general health indicators as well as to the Massachusetts Veterans Epidemiology Research and Information Center laboratory at VA Boston for where they were frozen −80 degrees Celsius for additional future biomarker testing.

## Results

### Sample Demographic and Clinical Characteristics - Descriptive Statistics

Only demographic and clinical characteristics are described in this report. As detailed in Table 4, this sample was predominantly white (66.7%), with an average age of 32.6 (SD = 7.1). Nearly one-third of the sample had either a bachelor's or advanced degree (29.4%) and 37.3% reported < $15,000 in annual household income and 31.4% reported an annual household income between $15,000 and $35,000.

**Table 4:** Sample demographics and descriptive characteristics.

**Full Sample (*n* = 51)**	***n* (%)/M ± SD**			
Age	32.6 ± 7.1			
Education				
High School/GED	9 (17.6%)			
Vocational/Technical Training	5 (9.8%)			
Some College Credit	15 (29.4%)			
Associate Degree	7 (13.7%)			
Bachelor's Degree	11 (21.6%)			
Post Grad Program	4 (7.8%)			
Race				
White	34 (66.7%)			
Black	10 (19.6%)			
Mixed Race/Other	7 (13.7%)			
**Non-IPV Trauma Exposure**	**Total prevalence**	**1–5 Times**	**6–20 Times**	**21+ Times**
Childhood Trauma	***n*** **(%)**	***n*** **(%)**	***n*** **(%)**	***n*** **(%)**
Sexual Assault	36 (70.6%)	21 (41.2%)	8 (15.6%)	7 (13.7%)
Physical Assault	24 (47.1%)	10 (19.6%)	3 (5.9%)	11 (21.6%)
Serious Accident	10 (19.6%)	10 (19.6%)	-	-
Exposure to Toxic Substance	2 (3.9%)	2 (3.9%)	-	-
Witnessed Sudden Violent Death	9 (17.7%)	9 (17.7%)	-	-
Sudden, Unexpected Death of Someone Close	15 (29.4%)	15 (29.4%)	-	-
Serious Injury, Harm, or Death You Caused	1 (2.0%)	1 (2.0%)	-	-
Captivity	4 (7.8%)	4 (7.8%)	-	-
Community Violence	4 (7.8%)	2 (3.9%)	2 (3.9%)	-
Adult Trauma	***n*** **(%)**	***n*** **(%)**	***n*** **(%)**	***n*** **(%)**
Sexual Assault	23 (45.1%)	18 (35.2%)	1 (2.0%)	4 (7.9%)
Physical Assault	15 (29.4%)	12 (23.5%)	2 (4.0%)	1 (2.0%)
Serious Accident	26 (51.0%)	25 (49.0%)	1 (2.0%)	-
Exposure to Toxic Substance	6 (11.8%)	4 (7.8%)	2 (3.9%)	-
Witnessed Sudden Violent Death	12 (23.5%)	10 (19.6%)	1 (2.0%)	1 (2.0%)
Sudden, Unexpected Death of Someone Close	35 (68.7%)	34 (66.7%)	1 (2.0%)	-
Serious Injury, Harm, or Death You Caused	5 (9.8%)	4 (7.8%)	1 (2.0%)	-
Captivity	11 (21.6%)	9 (17.7%)	1 (2.0%)	1 (2.0%)
Community Violence	19 (39.2%)	10 (19.6%)	6 (11.8%)	3 (5.9%)
**IPV Trauma Exposure**	***n*** **(%)/M ± SD**			
Any IPV	51 (100%)			
Physical	48 (94.1%)			
Sexual	36 (70.6%)			
Psychological/Emotional	49 (96.1%)			
Number of IPV relationships	2.6 ± 1.4			
Percent of adult life spent in IPV relationship	50.0% ± 32.1%			
Age at first IPV relationship experience	19.1 ± 5.8			
Time since last IPV abuse (Months)				
Physical assault	31.6 ± 37.0			
Sexual assault	51.7 ± 55.7			
Emotional abuse	31.6 ± 51.0			
Stalking	56.8 ± 70.3			
**Subconcussive Head Injury Information**	***n*** **(%)/M ± SD**			
Prevalence Subconcussive Head Injury (lifetime)	45 (88.2%)			
Number of Subconcussive HI (lifetime)^*^	2.8 ± 1.6			
Years since most recent HI (lifetime)^*^	6.7 ± 7.7			
Prevalence Subconcussive Head Injury (secondary to IPV)	39 (76.5%)			
Number of Subconcussive HI (secondary to IPV)^*^	1.8 ± 0.9			
Months Since Most Recent Subconcussive HI^*^	6.4 ± 6.5			
		***n*** **(%)**	***n*** **(%)**	***n*** **(%)**
Total Repetitive Blows	29.6 ± 41.7	**Low (0–10)**	**Medium (11–24)**	**High (≥25)**
		17 (33.3%)	17 (33.3%)	17 (33.3%)
**TBI Descriptive**	***n*** **(%)/M ± SD**			
Prevalence of TBI (lifetime)	27 (52.9%)			
Number of TBI (lifetime)^*^	1.9 ± 1.1			
Months Since Most Recent TBI (lifetime)^*^	9.1 ± 7.0			
Prevalence of TBI (secondary to IPV)	18 (35.3%)			
Number of TBI (secondary to IPV)^*^	1.3 ± 0.8			
Months Since Most Recent IPV-TBI^*^	10.1 ± 8.4			
Prevalence of Chocking/Anoxic event	16 (31.4%)			
Choking With LOC	4 (7.8%)			
Age of first TBI (by developmental stage)		**Age 0–12**	**Age >12–18**	**Age >18**
		5 (18.5%)	11 (40.7%)	21 (77.8%)
**PTSD**	**Current**	**Lifetime**		
PTSD diagnosis	41 (80.4%)	45 (88.2%)		
PTSD severity (CAPS)	35.1 ± 7.1	43.5 ± 9.6		
PTSD severity (PCL-5)	48.7 ± 12.7	-		
**Comorbid Psychiatric Disorders**	**Current**	**Lifetime**		
Major Depressive Disorder	11 (21.6%)	22 (43.1%)		
Panic Disorder	6 (11.8%)	1 (2.0%)		
Alcohol Use Disorder	9 (17.6%)	23 (45.1%)		
Cannabis Use Disorder	8 (15.7%)	12 (23.5%)		
Opioid Use Disorder	1 (2.0%)	7 (13.7%)		

* *Indicates values represent the M ± SD for the sub-group that endorsed experiencing the injury. n = 3 in the low repetitive blows group endorsed no history of head injury; GED, General Educational Development; IPV, Intimate Partner Violence; HI, Head Injury; TBI, Traumatic Brain Injury; PTSD, Post-Traumatic Stress Disorder; CAPS-5, Clinician Administered PTSD Scale for DSM-5; PCL-5, PTSD Checklist for DSM-5*.

### Trauma Exposures

The women in this sample reported high rates of trauma exposure during their lifetime. Regarding childhood trauma, exposures to various types of abuse are described in Table 4. Eighty percent of women reported sexual violence in their lifetimes (70.6% child, 45.1% adult) outside of an IPV relationship, and 56.9% reported non-IPV physical violence in their lifetimes (47.1% child, 29.4% adult). Further, on average, women reported spending half of their adult lives in an IPV relationship (50.0%) with a range from 16.7 to 95.5%. Within IPV relationships, all women reported psychological/emotional violence, nearly all reported physical violence (96.1%), and 72.6% reported sexual violence.

### PTSD

Given the rate of total lifetime trauma exposure, it is perhaps unsurprising that the sample also reported high levels of post-traumatic stress. Of the 51 women, 41 met full criteria for current PTSD (per CAPS-5 assessment) and 7 met for sub-threshold PTSD (within 1 SD below the mean CAPS-5 severity score).

### Subconcussive Head Injury and TBI

Of the 51 women, 88.2% reported experiencing one or more subconcussive head injuries in their lifetime (*M* = 2.5, *SD* = 1.8, *Range* = 1–8 incidents), with 76.5% reporting suffering one or more subconcussive head injuries within the context of IPV with an average of 1.8 (*SD* = 0.9, *Range* = 1–4) separate assaults resulting in a head injury (Table 4). Twenty-seven women met diagnostic criteria for at least one TBI (52.9%) within their lifetime and a third (35.3%) reported at least one TBI secondary to an assault by an intimate partner. Importantly, all reported TBIs were diagnosed as mild in severity. Nearly half of all TBIs reported occurred in adulthood (41.7%), with an extended period of time between the assessment and the most recent TBI (*M* = 9.1 years; *SD* = 7.0). Within those that reported TBI, the mean number of lifetime TBI was 1.93 (*SD* = 1.1, *Range* = 1–5) and the average number of TBIs that occurred within the context of an IPV relationship was 1.33 (*SD* =0.77, *Range* = 1–4). Notably, pertaining to IPV-related events, 16 (31.4%) of the women reported that they had been strangled; 4 of whom reported losing consciousness as a result, indicating a possible anoxic event. Exact duration of unconsciousness is unknown, but all LOC secondary to strangulation were described as brief.

The experience of repetitive, sub-concussive blows (not resulting in a diagnosable TBI) to the head, face, and neck within the context of an IPV relationship was pervasive in this sample. Forty-eight (94.1%) women reported experiencing one or more subconcussive head injuries within an IPV relationship and endorsed, on average, 29.6 (SD = 41.7) repetitive blows to their head, face, and neck (Table 4).

## Discussion

The overarching goal of this report was to provide a description of this unique multi-method assessment of the impact of head injuries on PTSD secondary to IPV in women. To our knowledge, this study represents the most comprehensive, multi-method assessment of PTSD and TBI among multiply traumatized women who have experienced violence in intimate relationships to date. Content and methodological experts in each area of inquiry were included in this multi-disciplinary team to ensure accurate assessments using state of the art methodology and gold standard clinician-administered instruments. A notable limitation in previous studies in the IPV literature is the lack of characterization of TBI and sub-concussive head injuries in the context of psychological distress. The current study aimed to address this deficit by developing clear methodology to assess and differentiate subconcussive head injury vs. TBI. This unique and innovative assessment is necessary to discriminate psychiatric symptoms from organic alterations in neurological structure and function due to head trauma.

A secondary goal of this report was to provide descriptive statistics of our sample given the relative paucity of studies in the IPV population and the lack of consensus within the available body of literature. Although research on the impact of TBI on PTSD has proliferated in recent years, the vast majority of these studies have been conducted in populations who have suffered these injuries through sports, falls, and combat-related concussive blasts. Research examining the impact of subconcussive head injury and TBI specifically in an IPV population has lagged far behind, despite the fact that the numbers of these types of injuries trump those in parallel populations combined. Generalizing the results of studies conducted in parallel head-injured populations to the IPV population is inadequate. Likewise, assessment of TBI and PTSD individually is far less effective than considering their interaction. Understanding the interactive effects of TBI and PTSD can only be accomplished through multi-modal methodology. This study represents the first in the IPV literature to include data collected via neuropsychological assessment, neuroimaging, and blood-based biomarkers while incorporating a full assessment battery measuring psychological distress and functional impairment, a comprehensive assessment of lifetime head injuries and exposure to traumatic events, and a full psychiatric history assessed via gold standard clinician-administered interviews.

Studies that have assessed head injuries in IPV samples have varied widely in their reports of the prevalence of TBI, in part due to significant inconsistencies in methodological approaches to TBI assessment and diagnosis (surveys, self-report, and clinical interviews). The accurate diagnosis of TBI is a critical first step in understanding the extent of this type of injury in the IPV population and its impact on recovery from PTSD. Toward this end, we modified the BAT-L, a semi-structured interview used to diagnose combat-related TBI in post 9/11 Veterans with demonstrated psychometric properties (23). This interview has several strengths including its ability to differentiate symptoms that are better attributable to psychological distress such as dissociation, confusion, and disorientation from symptoms of TBI such as AMS, PTA, and LOC. The BAT-L was modified to include IPV-specific probes designed to query the unique experiences and injuries of IPV survivors. The ability to differentiate subconcussive blows from TBIs within and outside of the context of IPV is a strength of the BAT-L/IPV [see (38) for more detail]. Using this comprehensive diagnostic assessment, we found lower rates of TBI than reported in several previous studies (Range: 28–100%; 6, 14, 25, 35). That said, 35.3% of our sample described a blunt force IPV-related injury that met criteria for a TBI and 76.5% reported one or more subconcussive head injuries secondary to an IPV-related assault. The BAT-L/IPV also queries injuries related to strangulation given the prevalence of this type of experience in IPV and its potential for brain injury. Approximately one-third of study participants reported strangulation severe enough to cause physiological disruption with ~8% of those incidents resulting in LOC. Importantly, the BAT-L/IPV also assesses severe, non-IPV head injuries across the lifetime. We detected a lifetime history of TBIs in half of the sample and a history of subconcussive blows in 88% of participants. This type of comprehensive assessment of lifetime head injury is critical in understanding the cumulative contribution of brain injury to participants' current clinical presentation.

The TRACTS assessment battery (93) was also expanded to assess the context in which IPV occurs as well as the individual's history of exposure to trauma that pre-dated the IPV relationship(s). Expansions included a number of self-report measures specific to IPV (see Table 1) and a clinician-administered IPV Trauma Interview developed specifically for this population (60). Consistent with previous research, participants reported complex histories of exposures to traumatic events throughout childhood and multiple exposures to non-IPV adult traumas. With respect to IPV, nearly all (94%) of the participants reported physical assaults, emotional abuse (96%), and sexual assaults (71%). The full complexity of participants' trauma history is apparent given the high rates of childhood abuse and early age of first IPV experience (on average, participants were 19 years old when first assaulted by an intimate partner). Multiple IPV relationships were common as, on average, women reported 2–3 different relationships with an average of 50% of adult lives being spent in a violent relationship. Understanding the full scope and breadth of exposure to trauma is a critical first step in diagnosing PTSD. Choosing the index trauma (worst event) on which to anchor the PTSD diagnosis from this complex array of different traumas can be clinically challenging and requires this level of assessment.

Utilization of gold standard, clinician-administered diagnostic instruments such as the CAPS-5 (59) and SCID-5 (81) to arrive at accurate lifetime and current psychiatric diagnoses secondary to the index trauma is a strength of this study. Study results revealed high rates of full diagnoses of current PTSD (80%) and co-occurring disorders such as major depression, panic disorder, and alcohol and substance use disorders. These rates of psychopathology were not surprising given that meeting clinical cutoffs for the PTSD screener was a necessary criteria for inclusion in the study. In sum, accurate diagnoses of TBI, detection of subconcussive blows, accurate diagnoses of PTSD and the full range of comorbid psychiatric disorders, and a complete assessment of lifetime trauma history including both IPV and non-IPV related traumas set the backdrop for future planned study hypotheses incorporating imaging, neuropsychological and blood-based biomarker data.

Data-sharing is another important advance in this area of research. With the informed consent of research participants, data collected from this sample will be incorporated into the larger TRACTS Data Repository (93). This repository houses longitudinal data from a cohort of over 500 post 9/11 deployed or scheduled-to-be deployed service members. An ongoing study, the TRACTS cohort consists of an ~90% male, ~75% White, and 100% military sample. Incorporating this data from a female sample of survivors of IPV will allow for future planned comparisons across genders and trauma types. Careful replication of the assessment battery developed by the TRACTS team allows for this seamless integration of data from female survivors of IPV.

### Limitations

This study is not without limitations. First, the design is retrospective and cross-sectional in nature. As such, the results of the study cannot be used to determine a cause-and-effect relationship; moreover, it may be difficult to determine whether some of the outcomes of interest followed exposure to head injury or vice versa. To address this limitation, the study team relied on both semi-structured interviews and self-report measures that are specifically designed to map timelines of injury and related symptoms. Additionally, participants that did not screen for probable PTSD cutoff scores were excluded from the study (*N* = 24) which limits the variance in PTSD symptomatology and may affect our ability to discriminate between TBI-related and PTSD-related neuropsychiatric symptoms following head injuries of IPV survivors. Self-report measurement has inherent limitations of its own, including potential lack of insight or the unintentional or intentional misrepresentation of experiences. To reduce these biases, all major outcomes of interest including head trauma and psychopathology were assessed with gold standard, clinician-administered interviews. However, it should also be noted that the interviews required participants to retrospectively report data that is difficult to recall such as length of time spent unconscious and/or amnesia. While the forensic approach used in the BAT-L/IPV is considered state-of-the-art methodology in collecting this data retrospectively, the lack of an accurate medical record collected at the time of the injury is a limitation. Finally, this study focused on women survivors of IPV and results will not be generalized beyond that population.

### Future Directions

This study has confirmed that the incidence of head injury is substantial and that further research is warranted to understand the impact of this type of injury on recovery from both physical and mental injury. In planned future publications, we will used the methods described in this paper to characterize the relative contributions of TBI and/or subconcussive head injury to psychiatric, psychological, and functional outcomes in this population with the ultimate goal of identifying novel targets for intervention and enhancing the overall effectiveness of current, single modal treatment strategies.

## Data Availability Statement

The datasets presented in this article are not readily available because the data are owned by the Boston Veteran Affairs Healthcare System and are subject to oversight by the VA. Requests to access the datasets should be directed to tara.galovski@va.gov.

## Ethics Statement

The studies involving human participants were reviewed and approved by Institutional Review Boards (IRB) at University of Missouri - St. Louis (IRB #1216580), Washington University in St. Louis (IRB #201810061), and VA Boston Healthcare System (Research and Development #10521). The patients/participants provided their written informed consent to participate in this study.

## Author Contributions

TG, KI, KW, and SK took the lead in writing the manuscript. TG conceived of the present idea and conceptual development the study. TG developed and led the study throughout implementation to manuscript preparation, supervising the project throughout. KI contributed significantly to the design of the research and the focus of the manuscript. KW managed the implementation of the study and coordination between research teams. CF contributed in the conceptualization of main outcome and how to interpret head injury vs. traumatic brain injury. AC processed the data and performed data analyses and prepared analytic tables for the current manuscript. JF supervised AC and assisted in data analyses and developed the data system and syntax used. DS and RM designed to original protocol that we replicated, modified, and expanded for our female intimate partner survivor sample. CF and RM also assisted in Figure and Table development. All authors discussed the development of the current protocol, and contributed by providing critical feedback which helped shape the research, analysis and manuscript.

## Conflict of Interest

The authors declare that the research was conducted in the absence of any commercial or financial relationships that could be construed as a potential conflict of interest.
